# Nutritional Quality of Plant-Based Drinks Sold in Italy: The Food Labelling of Italian Products (FLIP) Study

**DOI:** 10.3390/foods9050682

**Published:** 2020-05-25

**Authors:** Donato Angelino, Alice Rosi, Giorgia Vici, Marika Dello Russo, Nicoletta Pellegrini, Daniela Martini, on behalf of the SINU Young Working Group

**Affiliations:** 1 Faculty of Bioscience and Technology for Food, Agriculture and Environment, University of Teramo, 64100 Teramo, Italy; dangelino@unite.it; 2 Department of Food and Drug, University of Parma, 43100 Parma, Italy; alice.rosi@unipr.it; 3 School of Biosciences and Veterinary Medicine, University of Camerino, 62032 Camerino, Italy; giorgia.vici@unicam.it; 4 Institute of Food Sciences, National Research Council, 83100 Avellino, Italy; marika.dellorusso@isa.cnr.it; 5 Department of Agricultural, Food, Environmental and Animal Sciences, University of Udine, 33100 Udine, Italy; 6 Department of Food, Environmental and Nutritional Sciences (DeFENS), Università degli Studi di Milano, 20122 Milan, Italy; daniela.martini@unimi.it

**Keywords:** plant-based drinks, milk alternatives, nutrition declaration, nutritional values, food labeling

## Abstract

Plant-based drinks represent a heterogeneous class of beverages, made from several vegetal sources, with a market rapidly expanding around the world. These beverages are mainly drunk in the replacement of milk. Thus, aims of the present study were to: (i) evaluate the nutritional declaration of 330 plant-based drinks currently available on the Italian market; (ii) compare their nutrition facts based on type, presence or not of organic certification and nutrition (NC) or health claims (HC), and of specific claims (“no added sugars” and “source of calcium”); (iii) compare their nutrition composition with cow’s milk. A high variability in terms of nutrient profile among products was observed. Limited difference was found between products belonging to both organic and NC categories, while products carrying HC showed lower energy, carbohydrates, sugar, and higher protein contents than the related counterparts. Compared to cow’s milk, plant-based drinks showed differences in terms of nutrient profile, mostly regarding the lower protein content (except for soy drinks). Overall, due to the variability, findings from the present survey show that plant-based drinks sold in Italy cannot be considered *tout court* as milk substitutes and support the importance of improving knowledge towards food labeling to make conscious food choices.

## 1. Introduction

Plant-based drinks, also improperly called “vegetal milks,” represent a wide range of beverages that can be obtained by extracting in water a variety of plant ingredients, such as cereals (e.g., rice, oat, corn, spelt), pseudo-cereals (e.g., quinoa, amaranth), legumes (e.g., soy, lupin, chickpea), nuts (e.g., almond, hazelnut, peanut, walnut, pistachio), or seeds (e.g., flax, sesame, sunflower) [1,2].

One of the most common technologies to formulate plant-based beverages is based on the isolation of natural oil bodies from plant sources, which simulate milk fat globules that are compounds responsible for the creamy appearance [3]. To obtain these beverages from oil bodies, the plant material is soaked in water, mechanically grounded, blanched to turn off endogenous enzymes, centrifuged, thermally treated for microbiological safety, voluntarily fortified, and then packaged. In other alternative processes, emulsifiers and additives can be introduced [3]. As plant-based drinks are colloidal systems, they do not contain only fat globules, but also solid particles from raw materials, proteins, and starch granules. The presence of all these compounds threatens the stability of plant-based beverages which are consequently subjected to particle sedimentation [4]. In order to ameliorate stability, texture, and sensory attributes without affecting nutritional properties, innovative technologies involving specific combinations of pressure and temperature, e.g., high-hydrostatic-pressure processing and high-pressure homogenization, are used [5].

In the last years, there has been an increasing demand for non-dairy alternatives, which nowadays represent a wide segment for the food market [4,6]. The market for plant-based beverages is indeed constantly expanding, being projected to reach revenues of more than 38 billion USD by 2024 thanks to a current annual growth rate of over 14% during the forecast period between 2018 and 2024 [7]. Among non-dairy beverages, soy drinks dominate the market in the Western world with a global market size of 7.30 billion USD in 2018 [7]. Additionally, in Italy, according to data from the Italian Statistical Office (www.istat.it/en/) in 2016, the market of plant-based drinks was growing: in details, while soy drinks growth with a tendency of +2%, beverages based on other ingredients, such as almond, hazelnut, oats and coconut, growth in a volume of +75.1%, despite a ~30% higher price than soy drinks [8].

A comprehensive review by Sethi et al. [4] highlighted that this trend can be due to several different reasons, mostly related to health and nutrition concerns, that lead to a shift towards a plant-based diet. Among these, plant-based drinks are often consumed by individuals with lactose intolerance, a condition widespread in most of the world, with a prevalence ranging from 28% in Europe to 64%–70% in the Middle East and Asia [9]. More, plant-based drinks have been suggested for people with hypercholesterolemia or suffering of heart pathologies, being, for instance, these beverages free of animal fats [4,10] as well as for reaching the goal to consume fewer animal products [11].

As a result of the reasons listed above, the market is focusing on the production of innovative plant-based alternatives aimed to fulfill customer demands. In the meanwhile, the scientific community is investigating the innovative processing that can improve the nutritional value, the bioavailability of food components, and the sensory acceptability of plant-based beverages [5,10,12,13]. Moreover, researches are also focusing on the health effects of these products [14,15,16].

Aims of the present work were (i) to investigate the nutrition facts of plant-based beverages as declared on their food labels; (ii) to compare the energy and nutrient content of the products, classified for the type of vegetable source as well as for the presence or absence of nutrition claims (NC) or health claims (HC), or for being organic or conventional beverages); and (iii) to compare the nutritional values of plant-based beverages to those of cow milk.

This work is part of Food Labelling of Italian Products (FLIP) Study that aims at systematically investigating the overall quality of the pre-packed foods of the most important food groups and related categories sold on the Italian market.

## 2. Materials and Methods

### 2.1. The Selection of Food Product on Online Stores

Plant-based drinks considered for this work were selected from the major retailers selling products in Italy also in the e-commerce mode (Auchan, Bennet, Carrefour, Conad, Coop Italia, Crai, Despar, Esselunga, Il Gigante, Iper, Pam Panorama, Selex, Sidis), as already described in a previous manuscript [17]. Due to the characteristics of milk alternatives, three additional online retailers (Naturasì, Macrolibrarsi, and Sorgente Natura) were included.

Information was searched and collected online from January 2019 until December 2019. The following inclusion criteria were considered for the selection of products: (i) the availability of the item in at least one online shop; and (ii) the availability of all the data to be retrieved. All the items present in the online shops were considered eligible for data extraction.

The exclusion criteria for product selection were: (i) incomplete images of all the sides of the pack; (ii) unclear images of nutrition facts and/or list of ingredients; (iii) unavailability of the products in all the selected online stores during the entire data collection phase.

### 2.2. Data Extraction

As described elsewhere [17], the images of all the sides of the packaging were collected for all the included products, in order to retrieve the following regulated (mandatory) information as listed in the Council Regulation (EU) 1169/2011 [18]: company name, brand name, descriptive name, energy (kcal/100 mL), total fat (g/100 mL), saturates (g/100 mL), carbohydrate (g/100 mL), sugars (g/100 mL), protein (g/100 mL), and salt (g/100 mL). Furthermore, when available other information, i.e., the calcium content (mg/100 mL), the presence of a claim (i.e., presence or absence of at least one NC and of at least one HC as defined by the Council Regulation (EC) 1924/2006 [19]), the presence of the claim “no added sugars” and “source of calcium,” and the presence of the organic declaration (as defined by the Council Regulation (EC) No. 834/2007 [20]) was collected.

The precision of the extracted data was double-checked by two researchers (DA and GV) and inaccuracies were solved through secondary extractions (AR).

All the data was collected in a dataset and items were sub-grouped for specific comparisons taking into account (i) reported descriptive name, (ii) organic/not organic, (iii) presence or absence of NC declaration, (iv) presence or absence of specific NC (“with no added sugars” and “source of calcium” claim); (v) presence or absence of HC declaration. Based on the descriptive name, plant-based drinks were classified in 6 types: (i) soy drinks; (ii) rice drinks; (iii) almond drinks; (iv) oat drinks; (v) blends (≥2 plant-based ingredients); (vi) others (e.g., coconut, hazelnut, spelt, walnut, cashew drinks).

### 2.3. Statistical Analysis

For plant-based drinks, the statistical analysis was performed through the IBM SPSS statistics for Macintosh Version 26.0 (Armonk, NY, USA: IBM Corp.) with the significant level set at *p* < 0.05. The normality of data distribution was firstly verified through the Kolmogorov-Smirnov test and rejected. Variables were expressed as median (interquartile range). The Kruskal-Wallis non-parametric one-way ANOVA for independent samples with multiple pairwise comparisons was used to explore differences in energy and nutrient contents per 100 mL among types, while the Mann-Whitney non-parametric test for two independent samples was applied for differences between NC categories and HC categories.

To better explore variability among plant-based drink types and bring out a pattern in their nutritional composition, a Principal Component Analysis (PCA) with varimax rotation was executed taking into account energy and nutrient contents per 100 mL of soy, rice, almond and oat drinks. Blends and other products were excluded from this analysis to avoid the high variability linked to blended beverages or other products grouped in a single additional type.

Last, nutrition declaration of plant-based drinks was compared with the nutritional values for pasteurized cow’s milk and pasteurized skimmed cow’s milk, using data from the Food Composition Database for Epidemiological Studies in Italy [21].

## 3. Results

A total of 345 plant-based drinks were selected from the online retailers. Of these, 330 products were kept after removing the products that failed to meet the inclusion criteria established for this work. Products were mostly soy (25%) and rice (22%) drinks, followed by blended (18%), oat (12%), and almond (10%) drinks. In addition, 13% of products were grouped as “others,” such as coconut, hazelnut, spelt, walnut, cashew drinks, being less represented. In general, the majority of the selected plant-based drinks (74%) were organic products. Considering the presence of claims on the beverage package, 87% of products had at least an NC while only 16% reported at least one HC and 15% both nutrition and health claims. Among products with a nutrition claim, 183 (55% of the total products) had a claim referred to sugars (mainly “with no added sugars,” n = 178) and 125 (38% of the total products) to the calcium content (“source of calcium”). Regarding health claims, the most frequently reported claims were related to the role of calcium and vitamin D on bone health (n = 25 and 23, respectively) and the role of vitamins of the B group on fatigue (n = 23) [22].

### 3.1. Nutritional Composition of Plant-Based Drinks

The mandatory nutrition information indicated by the Council Regulation (EU) no. 1169/2011 [18] is reported for all plant-based drink types and for organic, NC, HC, “no added sugar” and “source of calcium” groups in Table 1.

The median energy content of the analyzed plant-based drinks was 50 (40–59) kcal/100 mL and it differed among product types (*p* < 0.01). It ranged between 57 (54–61) kcal/100 mL of rice drinks and 38 (26–46) kcal/100 mL of almond drinks, with rice drinks and blends having a higher energy value than the other product types. Similarly, differences among product types were observed for all nutrients (*p* < 0.01 for all), except for salt content. Rice drinks and blended beverages had the highest carbohydrate and sugar content, while soy and almond drinks contained the lowest. On the contrary, rice and oat had the lowest amount of total fats in comparison with other beverages. Moreover, soy drinks presented the highest quantities of saturated fats and proteins. Comparing organic and non-organic plant-based drinks, the former had a higher amount of energy (*p* = 0.001), total carbohydrates (*p* < 0.001), and sugars (*p* = 0.022), and a lower content of protein (*p* = 0.008) and salt (*p* < 0.001). When products with and without a nutrition claim were compared, only slight differences were observed for their lipid profile, having the products with a nutrition claim a lower amount of total fats and saturates (*p* < 0.01 and *p* = 0.022, respectively). Focusing on specific nutrition claims, products with the “no added sugars” claim had a lower content of total fat (*p* < 0.01), saturates (*p* < 0.01), protein (*p* < 0.01), and salt (*p* = 0.017), while they had a higher amount of total carbohydrates (*p* < 0.01) and similar content of sugar as those without this claim. “Source of calcium” products had a lower amount of energy (*p* = 0.004), total carbohydrates (*p* = 0.003) and sugars (*p* = 0.008), and a higher quantity of salt (*p* < 0.001) than plant-based drinks without the “source of calcium” claim. Considering products with and without a health claim, differences were observed for energy content (*p* < 0.01), total carbohydrates (*p* < 0.01), and sugars (*p* < 0.01), which were higher for products not bearing any health claim. On the contrary, products with a health claim showed a higher content of protein (*p* = 0.043) and salt (*p* = 0.001).

### 3.2. Inter-Product Variability of the Nutritional Composition of Plant-Based Drinks

**Figure 1:**
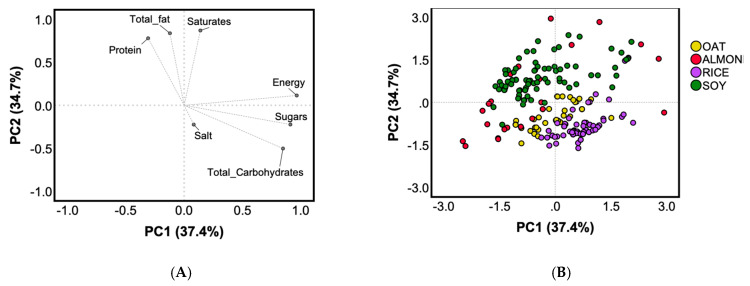
Principal component analysis (PCA) based on the nutritional composition of products belonging to soy, rice, almond or oat drinks (energy (kcal/100 mL), total fat (g/100 mL), saturates (g/100 mL), carbohydrate (g/100 mL), sugars (g/100 mL), protein (g/100 mL), and salt (g/100 mL)). Loading plots (**A**) of principal component (PC) 1 versus PC2; score plots (**B**) of the nutrition composition for each plant-based drink analyzed from PC1 and PC2.

The variability in the nutritional profile among the main types of plant-based drinks (i.e., oat, almond, rice, soy) was described by two principal components (PCs) (Figure 1).

These two PCs explained 72% of the total variability among products, respectively 37% (PC1) and 35% (PC2) (Figure 1A). Energy, total carbohydrate, and sugar contents positively loaded PC1, while PC2 showed positive loadings for total fat, saturated fat, and protein amounts. Salt contribution was negligible on both PCs. A high inter-product variability was observed for almond drinks, while soy, rice, and oat drinks clustered better. Due to their ingredients, the two cereal-based types (rice and oat drinks) were characterized by a high amount of total carbohydrates, sugars, and energy, while soy drinks by a high protein, total fat, and saturated fat contents (Figure 1B).

### 3.3. Comparison of the Nutritional Composition of Plant-Based Drinks with Cow Milk

**Figure 2:**
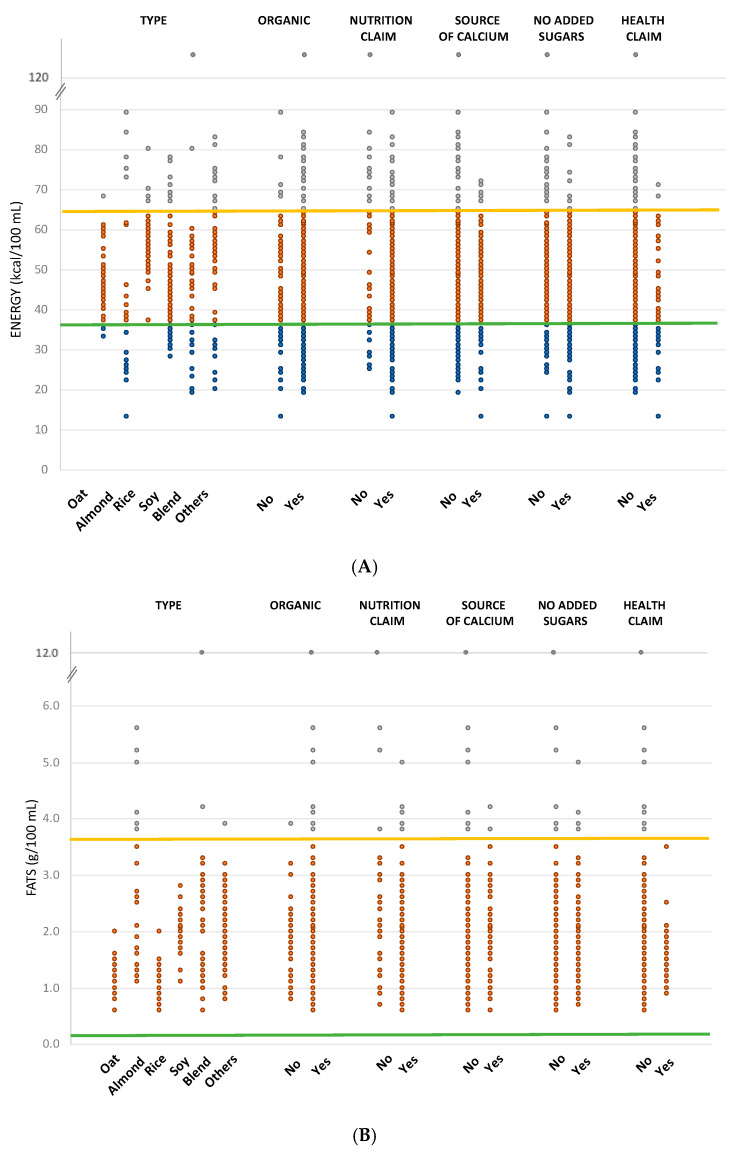

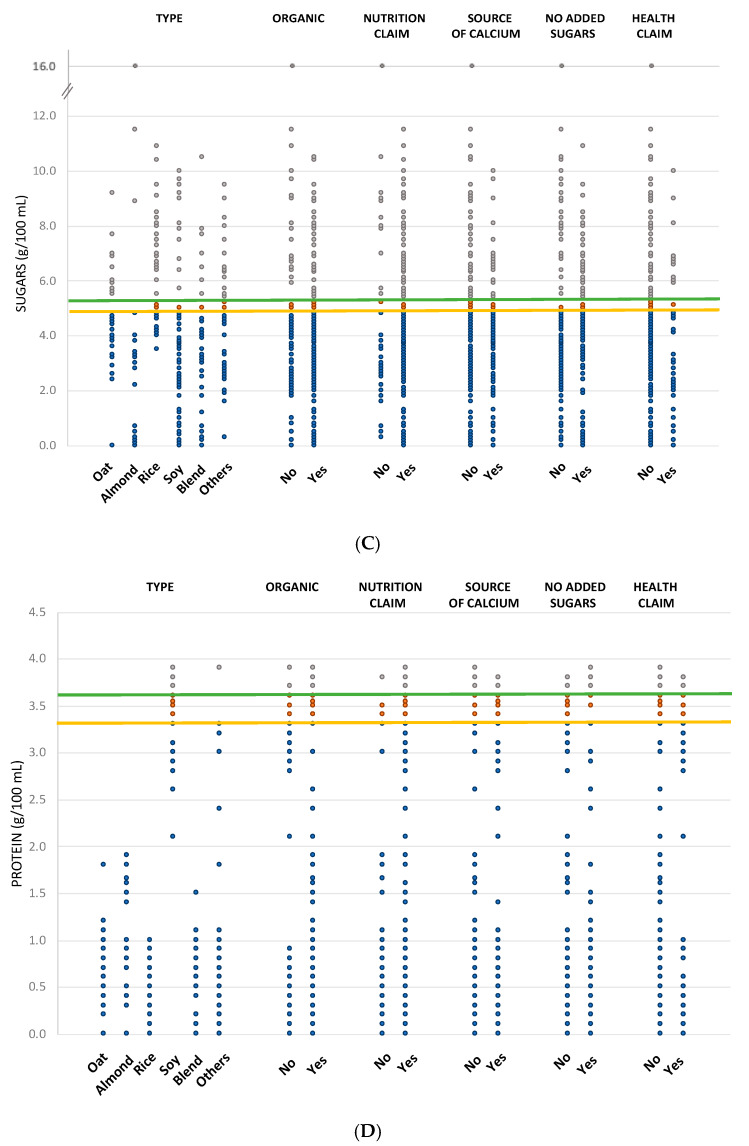
Energy (**A**), total fat (**B**), sugar (**C**), and protein (**D**) content of the analyzed plant-based drinks grouped by product types, organic or non-organic, having or not nutrition claim, health claim, “no added sugar” claim, and “source of calcium” claim. The orange line represents the value of regular milk, while the green line the value of skimmed milk. Blue, orange, and grey dots represent the referring values of each product lower, within, or higher than the reference values.

Plant-based drinks were compared to the nutritional composition of pasteurized whole cow milk and pasteurized skimmed cow milk. Comparisons for the most relevant nutrients (i.e., energy, fats, sugar, and protein) are shown in Figure 2A–D.

Considering energy, 15% of products had an energy content lower than that of skimmed milk (36 kcal/100 mL), 72% fell between the energy value of skimmed and regular milk, and 13% had a higher content of regular milk (64 kcal/100 mL). Among product types (Figure 2A), the majority of oat (90%), rice (89%), soy (76%), other (65%) and blended (54%) drinks had an energy content between the two reference values, while almond drinks were mainly split between products with energy content lower than the one of skimmed milk (44%), and products with energy content between skimmed and regular milk (41%). The majority of organic or non-organic products (72% for both), products with a nutrition claim (75%), with or without a health claim (66% and 73%, respectively), with or without “with no added sugars” claim (76% and 69%, respectively) and with or without “source of calcium” claim (76% and 70%, respectively) showed energy amount between the two reference milk products (Figure 2A). No plant-based drinks had a total fat content lower than skimmed milk (0.2 g/100 mL), while only 3% had a total fat amount higher than the regular milk (3.6 g/100 mL). Among product types, 100% of products belonging to oat, rice, and soy drinks had a total fat value between the two reference amounts, while 22% of almond drinks had a higher total fat content than regular milk (Figure 2B). The percentage of products having a total fat content between the ones of skimmed milk and regular milk was very similar between organic (96%) and non-organic (99%) products and between products with and without claims (ranging between 96% of products without a nutrition claim and 100% of products with a health claim), with or without “with no added sugars” claim (98% and 96%, respectively) and with or without “source of calcium” claim (97% for both) (Figure 2B). The sugar content of skimmed and regular milk (5.3 and 4.9 g/100 mL, respectively) is quite similar, thus only a few products (3%) fell within this range, while 73% had a sugar content lower than regular milk.

Coming to product types, almond, soy, and other drinks presented products with a sugar content lower than the regular milk (91%, 81%, and 72%, respectively), oat and blended drinks were equally distributed between products with sugar content lower than regular milk (58% for oat drinks and 44% for blends) and higher than skimmed milk (43% for oat drinks and 47% for blends), while 68% of rice drinks had a sugar amount higher than skimmed milk (Figure 2C). The sugar content of the majority of non-organic products (71%) was lower than the one of regular milk, while organic drinks were distributed between products with sugar amount lower than regular milk (55%) and with a higher sugar amount than skimmed milk (41%) (Figure 2C). The majority of products having a claim had less sugar than regular milk, ranging between 59% of products with a nutrition claim and 74% of drinks with a health claim (Figure 2C). Similarly, products with (52%) and without (70%) the “source of calcium” claim had sugar content lower than skimmed milk (Figure 2C). Considering products on the basis of the “with no added sugars” claim, 67% of plant-based drinks without the claim had a sugar amount lower than skimmed milk but only 52% of products with the claim had an amount lower than skimmed milk, while for 43% of products the sugar content was higher than the one of regular milk (Figure 2C). The protein amount of skimmed and regular milk is very similar (3.6 and 3.3 g/100 mL, respectively) thus only 7% of products fell within the reference values, while 88% of plant-based drinks had a lower content. Analyzing product types, all had a lower content than both regular and skimmed milk, except soy drinks, for which 18% of products were found to have a protein amount higher than skimmed milk, and 29% within regular and skimmed milk (Figure 2D).

Among organic categories and NC and HC categories, products with protein values higher than skimmed milk ranged between 2% for drinks without a nutrition claim and 7% for drinks not having the “source of calcium” claim (Figure 2D). Last, median calcium amount of “source of calcium” products (120 mg/100 mL) was found to be very similar to the one of both skimmed and regular milk (125 and 119 mg/100 mL, respectively), while calcium amount of products without this nutrition claim was unknown since was not reported on drinks’ nutritional labels.

## 4. Discussion

To the best of our knowledge, this is the first survey aiming at comprehensively evaluate the nutritional quality of plant-based drinks currently sold on the Italian market, as well as at comparing their nutritional values with those of cow’s milk. It is recognized worldwide that these beverages are mainly drunk in replacement of milk and its derivatives [15]. The first intriguing finding got from this study regards the number of items present on the shelves—330 different beverages—which might be a proxy of the increasing interest in these drinks by the Italian population. This Italian trend is in line with the one of other countries where the market of these products is rapidly growing [15]. For instance, in the US nondairy milk market had significant growth of 50% in 2018, while in the Asia Pacific area the compound annual growth rate was about 15% [7]. Regarding the nutrient profile, wide variability in terms of energy as well as macronutrient contents has been found, as among the products belonging to the same type as by comparing products from different vegetable sources. In detail, it has been found that rice- and oat-based products are richer in total carbohydrates than the other ones, while soy- and almond-derived products in total fats. As expected, soy-based beverages had the highest protein content, with a median value ~3-fold higher than the other ones. Salt values were particularly low in all the beverages and negligible differences were found among them. On the whole, the data of energy, macronutrients, and salt collected from the 330 items on the Italian market are in line with the ones of products considered in German [16], Spanish [5], and Indian [4] surveys. Interestingly, surveys from North American products (USA [3] and Canada [10]) reported a content of total fats and carbohydrates in “mil -alternatives” that is, on average, higher than that in the Italian ones.

Beside this, it is worth noting that plant-based drinks are considered as an alternative to milk probably because of the similar appearance in terms of liquid density and white color [4]. However, one of the major concerns of considering these products alternative to milk is related to the differences in terms of nutritional profile that have been shown to potentially lead to a reduced intake of certain macro and micronutrients [23]. In the present survey, the first and most important aspect worth being underlined is the difference in terms of protein amount contained in all the considered plant-based drinks, when compared to cow’s milk. Moreover, among the plant-based beverages, the organic ones had a median protein content four times lower than their non-organic counterparts. Data from the Food Composition Database for Epidemiological Studies in Italy [21] show an average protein content of ~3.5 g/100 mL for both regular and semi-skimmed cow’s milk. Our data reported that most of the products have a protein content lower than 1 g/100 mL, except for soy-based drinks, which had a median protein value of 3.3 g/100 mL, slightly higher than the findings previously reported [24]. However, despite the fact that it cannot be inferred by nutrition facts information and from our data, it is confirmed that, regardless of their protein content, the biological quality of plant-based proteins is lower than the animal one. In this term, exhaustive works [25,26] considered the protein quality of milk and plant-based beverages by means of the recently FAO developed Digestible Indispensable Amino Acid Score (DIAAS) [27]. Results showed a >100% DIAAS value for all the indispensable milk amino acids, a ~90% DIAAS for soy beverages, which are substantially lacking essential amino acids such as methionine, and a < 50% DIAAS for all the other considered vegetable source of proteins [25,26].

Nutritional differences between milk and plant-based beverages are not only relevant for the macronutrient amounts, but also for micronutrients. Milk—and dairy products—are the most relevant source of dietary calcium, with 120 mg/100 mL for cow’s milk [21]. Not only the amount but also the bioavailability of the calcium is high in milk where calcium ions are weakly linked to phosphorylated serine residues of casein. Conversely, in the plant-derived beverages, calcium is trapped with strong bounds by antinutritive components, e.g., phytates, oxalates, etc. [28]. In Italy, population reference intake for calcium is 1000 mg for both men and women (aged ≥ 18 years) and up to 1200 mg for postmenopausal women (aged ≥ 60 years) [29]. National surveys indicated a median value of 792 mg for adult males and 697 mg for adult females, and slightly higher for elderlies [30]. Being these values are far from the recommended values, it would be risky to consider plant-based beverages as substitutes for the daily portion of milk and, in turn, dairy products. More, milk is not only a good source of phosphorus, which bounds calcium to milk caseins, but also of iodine [31], an essential microelement for thyroid hormone synthesis, which is instead present in low amount in soy, rice, oat, and almond beverages unless added [32].

The present survey detailed that 77% of the items on the market boasted a nutrition claim. On the whole, when compared with beverages without nutritional claim, they did not show any significant nutritional quality difference, except for a slight reduction of total fat and saturates, not directly attributable to the claim. This finding partially supports the hypothesis that NC carried on the pack cannot be always considered a marker of a better overall nutritional quality of the product, in agreement with our previous FLIP study [17]. However, interesting results have been shown by considering some of the single claims. For example, by looking at beverages “with no added sugar,” no difference in terms of sugars—indeed an increased median value of total carbohydrates—has been found compared to beverages not boasting the claim. Plant-based drinks “source of calcium” were characterized, on median value, by lower energy, total carbohydrate, and sugar values than the regular ones. Interestingly, 88% of the beverages boasting the claim contained exactly 120 mg/100 mL of calcium, which is the average calcium content of the cow’s milk. This finding suggests the interest by the food industry in the functionalization of these products towards a similar profile—at least for calcium—than cow’s milk.

Although the beverages claiming a health effect accounted for only 16% of the total, it is intriguing to discuss their data with respect to other beverages. These beverages showed a lower energy content—mainly attributable to the lower amount of total carbohydrates and sugars—and higher protein values compared to products with no health claims. This is in agreement with studies reporting only a marginally better nutrition profile in foods boasting health claims compared to those without claims [33].

Finally, we found that 74% of the plant-based beverages have been certified as organic products, but this aspect seems not to substantially improve the nutritional quality of the drinks; on the contrary, they showed a double median value of total carbohydrates and higher sugars than the not-organic drinks. This aspect is in line with the one that emerged from our FLIP database concerning other food categories, confirming that the organic certification cannot be a proxy of better nutritional quality of these products compared to non-organic [34].

This study has some limitations. One is relative to the missing comparison of additional elements, such as vitamins, minerals, and fiber. Unfortunately, no assumptions can be done for these elements, for which values are not mandatory on the food pack, in agreement with the Council European Regulation (EU) no. 1169/2011 [18]. More, it cannot be estimated whether retailers update the online information of the products’ nutritional data, and this may partially limit the accuracy and the actuality of the data. However, we tried to ensure the use of up-to-date nutrition facts by comparing the different nutrition declarations when the same product was available in more than one retailer.

Finally, being the main focus of the study the analysis of the nutritional declaration, other aspects as the list of ingredients have not been fully analyzed. For some declarations, e.g., HC, the number of products bearing the claims or not were pretty different and this aspect might have affected the results. We were not able to review some sales channels, e.g., local shops or vending machines. This survey is a snapshot of what is effectively sold on the Italian market, and this is one of the main strengths of the study.

## 5. Conclusions

The present work investigated the nutritional quality of the most common plant-based drinks sold on the Italian market, pointing out a wide variability in terms of energy and macronutrient contents. This research further confirmed previous findings in relation to products boasting health or nutritional claims, as well as organic certification, which did not necessarily indicate a significant improvement in the nutritional quality of these beverages.

Plant-based drinks are commonly used as an alternative to milk and, for this reason, in the present research there was a focus also on the main differences between their nutritional profile and that of common cow’s milk. Results underlined that there is a large variability in terms of nutrition facts, mostly dependent on the plant ingredient used and to the fact of being or not fortified. In this scenario, it is worth noting that only one out of three plant-based drinks contained an amount of (added) calcium comparable to milk. Thus, plant-based beverages cannot be considered tout court as milk alternatives.

Overall, these data support that there is the need for education in reading and understanding the information declared on the food package in order to increase consumer knowledge about the nutritional quality of plant-based beverages as well as of all the other products. The customer’s intention to buy is also guided by their capability to focus and understand each piece of information present on packages which, in turn, is at the basis of their healthy choices.

## Figures and Tables

**Table 1:** Mandatory nutrition declaration across plant-based drink categories.

		Number of Items	Energy	Total Fat	Saturates	Total Carbohydrates	Sugars	Protein	Salt
			kJ/100 mL/ kcal/100 mL	g/100 mL	g/100 mL	g/100 mL	g/100 mL	g/100 mL	g/100 mL
Total Plant-based Drinks		330	210 (168–250) /50 (40–59)	1.6 (1.1–2.1)	0.3 (0.2–0.4)	7.7 (3.1–10.9)	4.4 (2.7–6.4)	0.7 (0.2–3.0)	0.10 (0.08–0.11)
Type	Oat	40	195 (176–214) /47 (42–51) ^b^	1.2 (1.1–1.5) ^b^	0.2 (0.2–0.3) ^b^	7.9 (7.0–9.0) ^b,c^	4.5 (3.6–6.0) ^b^	0.6 (0.4–0.9) ^b^	0.10 (0.09–0.10)
	Almond	32	160 (109–193) /38 (26–46) ^b^	2.3 (1.3–3.4) ^a^	0.2 (0.2–0.4) ^b^	3.0 (0.7–4.7) ^d,e^	3.0 (0.2–3.8) ^c^	0.8 (0.5–1.0) ^b^	0.10 (0.00–0.13)
	Rice	72	239 (227–256) /57 (54–61) ^a^	1.0 (1.0–1.1) ^b^	0.2 (0.2–0.2) ^b^	12.0 (10.5–13.0) ^a^	6.2 (5.0–7.4) ^a^	0.2 (0.0–0.4) ^c^	0.10 (0.09–0.10)
	Soy	84	185 (164–227) /44 (39–54) ^b^	2.0 (1.8–2.1) ^a^	0.3 (0.3–0.4) ^a^	3.0 (1.5–4.9) ^e^	2.6 (1.0–4.3) ^c^	3.3 (3.0–3.6) ^a^	0.10 (0.05–0.14)
	Blends	59	248 (206–281) /59 (49–67) ^a^	1.9 (1.2–2.4) ^a^	0.3 (0.2–0.7) ^a^	10.0 (5.0–12.1) ^b^	5.1 (3.4–6.5) ^a,b^	0.6 (0.3–0.8) ^b^	0.10 (0.08–0.10)
	Others	43	193 (151–223) /46 (36–53) ^b^	1.6 (1.1–2.6) ^a^	0.3 (0.1–1.1) ^a,b^	4.6 (2.2–10.0) ^c,d^	3.9 (2.7–5.5) ^b,c^	0.5 (0.2–0.8) ^b^	0.10 (0.07–0.12)
Organic	No	85	185 (164–227) /44 (39–54) ^b^	1.7 (1.1–2.0)	0.3 (0.2–0.4)	4.2 (2.8–8.3) ^b^	3.3 (2.6–5.9) ^b^	2.8 (0.3–3.3) ^a^	0.10 (0.10–0.14) ^a^
	Yes	245	223 (172–256) /53 (41–61) ^a^	1.5 (1.1–2.1)	0.3 (0.2–0.4)	9.0 (3.7–11.0) ^a^	4.8 (3.0–6.5) ^a^	0.6 (0.2–1.1) ^b^	0.10 (0.08–0.10) ^b^
Nutrition Claim	No	43	206 (160–294) /49 (38–70)	2.0 (1.3–2.5) ^a^	0.3 (0.2–0.6) ^a^	5.4 (3.2–11.0)	3.7 (2.7–8.0)	0.7 (0.3–1.1)	0.10 (0.04–0.13)
	Yes	287	210 (171–244) /50 (40–58)	1.5 (1.1–2.0) ^b^	0.3 (0.2–0.4) ^b^	7.9 (3.1–10.5)	4.5 (2.7–6.4)	0.7 (0.2–3.0)	0.10 (0.08–0.11)
“No Added Sugar” Claim	No	152	204 (168–255) /49 (40–61)	1.9 (1.3–2.2) ^a^	0.3 (0.2–0.4) ^a^	4.5 (3.0–9.6) ^b^	3.6 (2.6–6.9)	1.6 (0.5–3.3) ^a^	0.10 (0.08–0.13) ^a^
	Yes	178	214 (172–248) /51 (41–59)	1.3 (1.0–2.0) ^b^	0.2 (0.1–0.4) ^b^	9.7 (5.2–11.0) ^a^	4.9 (2.9–6.4)	0.5 (0.2–0.9) ^b^	0.10 (0.08–0.10) ^b^
“Source of Calcium” Claim	No	205	223 (170–256) /53 (41–61) ^a^	1.6 (1.1–2.2)	0.3 (0.2–0.4)	9.0 (3.4–11.0) ^a^	4.8 (2.9–6.6) ^a^	0.6 (0.3–1.0)	0.10 (0.08–0.10) ^b^
	Yes	125	202 (168–235) /48 (40–56) ^b^	1.6 (1.1–2.0)	0.2 (0.2–0.3)	5.4 (3.0–10.0) ^b^	3.9 (2.6–5.5) ^b^	0.9 (0.2–3.0)	0.10 (0.09–0.13) ^a^
Health Claim	No	277	218 (181–252) /52 (43–60) ^a^	1.6 (1.1–2.1)	0.3 (0.2–0.4)	8.4 (3.5–11.0) ^a^	4.6 (3.1–6.5) ^a^	0.6 (0.2–1.8) ^b^	0.10 (0.08–0.10) ^b^
	Yes	53	168 (147–218) /40 (35–52) ^b^	1.7 (1.1–1.9)	0.2 (0.2–0.3)	3.2 (2.5–7.9) ^b^	2.6 (2.0–5.1) ^b^	2.1 (0.3–3.3) ^a^	0.10 (0.09–0.15) ^a^

Values are median (25th–75th percentile). Different lowercase letters in the same column indicate significant differences among plant-based drink types (Kruskal–Wallis non-parametric test for independent samples with multiple pairwise comparisons, *p* < 0.05) and between organic, nutrition claim, health claim, “with no added sugars” claim, and “source of calcium” claim categories (Mann–Whitney non-parametric test, *p* < 0.05).

## SINU Young Working Group

Margherita Dall’Asta	Department of Animal Science, Food and Nutrition, Università Cattolica del Sacro Cuore, Piacenza, Italy
Stefania Moccia	Institute of Food Sciences, National Research Council, Avellino, Italy
Daniele Nucci	Veneto Institute of Oncology IOV-IRCCS, Padova, Italy
Gaetana Paolella	Department of Chemistry and Biology A. Zambelli, University of Salerno, Fisciano, Italy
Veronica Pignone	Department of Epidemiology and Prevention, IRCCS Neuromed, Pozzilli, Italy
Emilia Ruggiero	Department of Epidemiology and Prevention, IRCCS Neuromed, Pozzilli, Italy
Carmela Spagnuolo	Institute of Food Sciences, National Research Council, Avellino, Italy

## References

[1] Mäkinen O.E., Wanhalinna V., Zannini E., Arendt E.K. (2016). Foods for special dietary needs: Non-dairy plant-based milk substitutes and fermented dairy-type products. Crit. Rev. Food Sci. Nutr..

[2] Silva A.R.A., Silva M.M.N., Ribeiro B.D. (2020). Health issues and technological aspects of plant-based alternative milk. Food Res. Int..

[3] McClements D.J., Newman E., McClements I.F. (2019). Plant-based Milks: A review of the science underpinning their design, fabrication, and performance. Compr. Rev. Food Sci. Food Saf..

[4] Sethi S., Tyagi S.K., Anurag R.K. (2016). Plant-based milk alternatives an emerging segment of functional beverages: A review. J. Food Sci. Technol..

[5] Munekata P.E.S., Domínguez R., Budaraju S., Roselló-Soto E., Barba F.J., Mallikarjunan K., Roohinejad S., Lorenzo J.M. (2020). Effect of innovative food processing technologies on the physicochemical and nutritional properties and quality of non-dairy plant-based beverages. Foods.

[6] Plant Based Foods Association Explosive Growth in Dairy Alternatives Market Expected Through 2020, Study Finds. www.plantbasedfoods.org/explosive-growth-dairy-alterna-tives-market-expected-2020-study-finds/.

[7] Aritzon Non-Dairy Milk Market-Global Outlook and Forecast 2019–2024. https://www.marketresearch.com/Arizton-v4150/Non-Dairy-Milk-Global-Outlook-12287735/.

[8] Nielsen Holdings Italians Change Habits but Do Not Give Up Breakfast at Home. https://www.nielsen.com/it/it/insights/article/2017/italians-change-habits-but-do-not-give-up-breakfast-at-home/.

[9] Storhaug C.L., Fosse S.K., Fadnes L.T. (2017). Country, regional, and global estimates for lactose malabsorption in adults: A systematic review and meta-analysis. Lancet Gastroenterol. Hepatol..

[10] Vanga S.K., Raghavan V. (2018). How well do plant based alternatives fare nutritionally compared to cow’s milk?. J. Food Sci. Technol..

[11] McCarthy K.S., Parker M., Ameerally A., Drake S.L., Drake M.A. (2017). Drivers of choice for fluid milk versus plant-based alternatives: What are consumer perceptions of fluid milk?. J. Dairy Sci..

[12] Jeske S., Zannini E., Arendt E.K. (2018). Past, present and future: The strength of plant-based dairy substitutes based on gluten-free raw materials. Food Res. Int..

[13] Tangyu M., Muller J., Bolten C.J., Wittmann C. (2019). Fermentation of plant-based milk alternatives for improved flavour and nutritional value. Appl. Microbiol. Biotechnol..

[14] Jeske S., Zannini E., Arendt E.K. (2017). Evaluation of physicochemical and glycaemic properties of commercial plant-based milk substitutes. Plant Foods Hum. Nutr..

[15] Paul A.A., Kumar S., Kumar V., Sharma R. (2019). Milk Analog: Plant based alternatives to conventional milk, production, potential and health concerns. Crit. Rev. Food Sci. Nutr..

[16] Scholz-Ahrens K.E., Ahrens F., Barth C.A. (2020). Nutritional and health attributes of milk and milk imitations. Eur. J. Nutr..

[17] Angelino D., Rosi A., Dall’Asta M., Pellegrini N., Martini D. (2019). Evaluation of the nutritional quality of breakfast cereals sold on the italian market: The Food Labelling of Italian Products (FLIP) study. Nutrients.

[18] European Union Council (2011). European Union Council Regulation No 1169/2011 on the provision of food information to consumers. Off. J. Eur. Union.

[19] European Union Council (2006). European Union Council Regulation No 1924/2006 on nutrition and health claims made on foods. Off. J. Eur. Union.

[20] European Union Council (2007). European Union Council Regulation No 834/2007 on organic production and labelling of organic products and repealing Regulation (EEC) No 2092/91. Off. J. Eur. Union.

[21] Gnagnarella P., Salvini S., Parpinel M. Food Composition Database for Epidemiological Studies in Italy. http://www.bda-ieo.it/.

[22] European Union Council (2012). European Union Council Regulation No 432/2012 establishing a list of permitted health claims made on foods, other than those referring to the reduction of disease risk and to children’s development and health. Off. J. Eur. Union.

[23] Zhang Y.Y., Hughes J., Grafenauer S. (2020). Got mylk? The emerging role of Australian plant-based milk alternatives as a cow’s milk substitute. Nutrients.

[24] Chalupa-Krebzdak S., Long C.J., Bohrer B.M. (2018). Nutrient density and nutritional value of milk and plant-based milk alternatives. Int. Dairy J..

[25] Singhal S., Baker R.D., Baker S.S. (2017). A Comparison of the nutritional value of cow’s milk and nondairy beverages. J. Pediatr. Gastroenterol. Nutr..

[26] Rutherfurd S.M., Fanning A.C., Miller B.J., Moughan P.J. (2015). Protein digestibility-corrected amino acid scores and digestible indispensable amino acid scores differentially describe protein quality in growing male rats. J. Nutr..

[27] FAO Food and Nutrition Dietary Protein Quality Evaluation in Human Nutrition: Report of an FAO Expert Consultation. http://www.fao.org/ag/humannutrition/35978-02317b979a686a57aa4593304ffc17f06.pdf.

[28] Brody T. (1994). Nutritional Biochemistry.

[29] Italian Society of Human Nutrition (SINU) (2014). Livelli di Assunzione di Riferimento di Nutrienti ed Energia per la Popolazione Italiana.

[30] Sette S., Le Donne C., Piccinelli R., Arcella D., Turrini A., Leclercq C. (2011). The third Italian National Food Consumption Survey, INRAN-SCAI 2005–06-Part 1: Nutrient intakes in Italy. Nutr. Metab. Cardiovasc. Dis..

[31] Roseland J.M., Phillips K.M., Patterson K.Y., Pehrsson P.R., Bahadur R., Ershow A.G., Somanchi M. (2020). Large variability of iodine content in retail cow’s milk in the USA. Nutrients.

[32] Ma W., He X., Braverman L. (2016). Iodine content in milk alternatives. Thyroid.

[33] Kaur A., Scarborough P., Hieke S., Kusar A., Pravst I., Raats M., Rayner M. (2016). The nutritional quality of foods carrying health-related claims in Germany, the Netherlands, Spain, Slovenia and the United Kingdom. Eur. J. Clin. Nutr..

[34] Dall’Asta M., Angelino D., Pellegrini N., Martini D. (2020). The Nutritional quality of organic and conventional food products sold in Italy: Results from the food labelling of Italian products (FLIP) Study. Nutrients.

